# A method for preparation and cleaning of uniformly sized arsenopyrite particles

**DOI:** 10.1186/s12932-014-0014-9

**Published:** 2014-10-11

**Authors:** Hariprasad Parthasarathy, John P Baltrus, David A Dzombak, Athanasios K Karamalidis

**Affiliations:** 1 Department of Civil and Environmental Engineering, Carnegie Mellon University, Pittsburgh, 15213 PA USA; 2 U.S. Department of Energy, National Energy Technology Laboratory, Pittsburgh, 15236 PA USA

**Keywords:** Arsenopyrite, XPS, Mineral preparation, Surface cleaning, Oxidation

## Abstract

**Background:**

The oxidative dissolution of sulfide minerals, such as arsenopyrite (FeAsS), is of critical importance in many geochemical systems. A comprehensive understanding of their dissolution rates entails careful preparation of the mineral surface. Measurements of dissolution rates of arsenic from arsenopyrite are dependent on the size and degree of oxidation of its particles, among other factors. In this work, a method was developed for preparation and cleaning of arsenopyrite particles with size range of 15-250 μm. Four different cleaning methods were evaluated for effectiveness based on the removal of oxidized species of iron (Fe), arsenic (As) and sulfur (S) from the surface. The percentage oxidation of the surface was determined using X-ray photoelectron spectroscopy (XPS), and surface stoichiometry was measured using scanning electron microscopy - energy dispersive X-ray spectroscopy (SEM-EDS).

**Results:**

Results indicate that sonicating the arsenopyrite particles and then cleaning them with 12N HCl followed by 50% ethanol, and drying in nitrogen was the most effective method. This method was successful in greatly reducing the oxide species of Fe while completely removing oxides of As and S from the arsenopyrite surface.

**Conclusions:**

Although sonication and acid cleaning have been widely used for mineral preparation, the method described in this study can significantly reduce grain size heterogeneity as well as surface oxidation, which enables greater control in surface and dissolution experiments.

**Electronic supplementary material:**

The online version of this article (doi:10.1186/s12932-014-0014-9) contains supplementary material, which is available to authorized users.

## Background

Arsenopyrite (FeAsS (s)) is the most common arsenic (As) bearing pure phase mineral in the earth's crust. It is present in a variety of deposits such as hydrothermal, and magmatic systems and is an important reservoir of arsenic in the subsurface. Due to its common association with gold, it is often discarded as solid waste after gold extraction. The oxidation of arsenopyrite can release As into the environment which has potential environmental and health impacts [1].

A number of studies [2]-[5] have been conducted that investigate the kinetics of arsenopyrite dissolution with oxidants such as dissolved oxygen and iron, but there is significant variation in reported rates. One of the possible sources of this variation is the lack of a consistent mineral preparation procedure. Differences in mineral preparation can significantly affect grain size distribution as well as affect the extent of oxidation on the surface prior to conducting dissolution studies.

Previous research [3] indicates that grain sizes can exert significant control over oxidation and dissolution rates. McKibben et al. [3] determined that 150-250 μm was the most convenient grain size for arsenopyrite dissolution. Arsenopyrite is typically prepared by homogenous grinding of the sample in a mortar and pestle, and then dry sieved to obtain required size fractions. Fine particles have high specific surface areas and their presence in these fractions can cause exaggerated dissolution rates [6], as well as affect reproducibility of dissolution experiments. Typically, sonication of crushed mineral in ethanol or acetone has been used to remove fines from the surface of sulfide minerals [3],[7],[8].

Dissolution studies can be further complicated by the presence of oxidized species on the mineral surface, which can lead to erroneous initial rates [2]. Since the dissolution of arsenopyrite is oxidative in nature, oxidized species on the surface could drive subsequent dissolution of arsenopyrite. Acid cleaning has been used extensively in the literature as a method of cleaning sulfide minerals such as pyrite and arsenopyrite, but relies on indirect methods such as initial sulfate release to determine the extent of surface oxidation. Moses et al. [9] reported the use of a combination of boiling 6N HCl and acetone for surface cleaning of pyrite. The extent of oxidation was determined by monitoring the immediate release of SO_4_^2-^ into solution. In the case of arsenopyrite, McKibben at al. [3] reported the use of 1.8 N HNO_3_ for cleaning, while other groups [2],[4] did not report methods for surface oxide removal. Oxidized iron and arsenic were observed to be removed from the surface of arsenopyrite when it was immersed in an air-saturated acetic acid solution [10]. However, studies on the relative rates of elemental oxidation of arsenopyrite upon exposure to air have shown that As and Fe oxidize at rates faster than S [11],[12], suggesting that measurement of immediate release of sulfate into solution might not accurately reflect the extent of surface oxidation. As a result, there exists a need for direct evaluation of acid cleaning as a viable method for surface oxide removal on arsenopyrite prior to dissolution experiments. Typically, X-ray photoelectron spectroscopy (XPS) has been used to study surface oxidation on many minerals, but studies on arsenopyrite have been limited to vacuum fractured pristine surfaces and surfaces exposed to oxidants.

The objective of this study was to develop a reproducible and effective procedure to generate arsenopyrite particles of a uniform size fraction, free of surface oxides. The specific objectives were to (i) obtain arsenopyrite particles of the size fraction 150-250 μm by removing fines adhering to the surface, and (ii) clean the surface of crushed particles to remove oxide species of Fe, As and S, without altering the stoichiometry of the mineral. The effectiveness of HCl and acetic acid for cleaning the surface of arsenopyrite was evaluated, based on their reported use for this purpose in the literature. The resulting method developed for cleaning the arsenopyrite surface was verified through XPS, particle size distribution analysis, and scanning electron microscopy - energy dispersive X-ray spectroscopy (SEM-EDS).

## Results and discussion

### Particle size analysis

**Figure 1:**
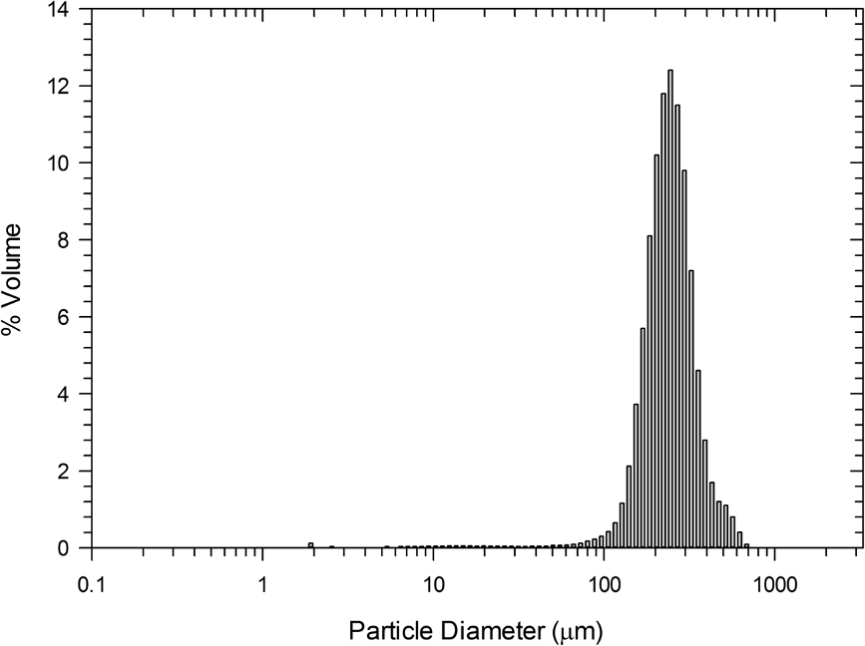
**Particle size distribution of prepared arsenopyrite particles expressed as volume %.**

The results of the particle size distribution are shown in Figure 1. The particles were normally distributed with a mean diameter of 208.9 μm and a standard deviation of 1.764 μm, and the median was 219.2 μm. This suggests that while the distribution was not entirely symmetric, a large fraction of particles was within the size range of 150-250 μm.

### XPS

#### Drying

The effects of two different drying protocols on the extent of arsenopyrite surface oxidation can be seen by examining the results of curve fitting the three elemental regions: As 3d, Fe 2p_3/2_, and S 2p in Table 1.

**Table 1:** **The effect of drying method on surface oxidation of arsenopyrite**

Drying method	% Oxidation on the surface		
	Fe	As	S
In air, 105°C	93.9	92.0	63.7
In air, 25°C	43.8	42.9	11.9

**Figure 2:**
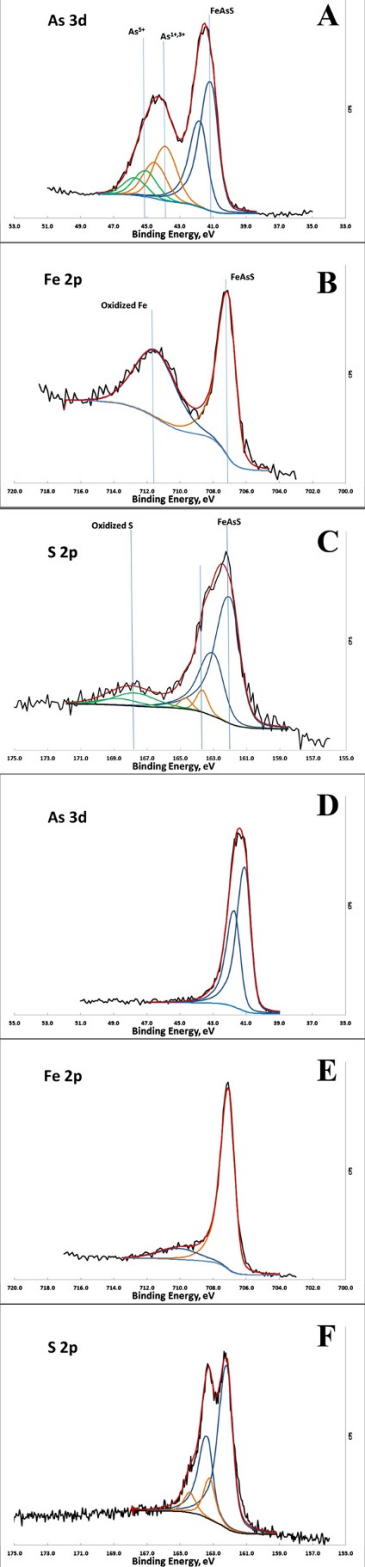
**XPS spectra depicting speciation of As, Fe, and S on arsenopyrite.** Representative curve fitted XPS spectra for **(A)** arsenic - As 3d, **(B)** iron- Fe 2p, and **(C)** sulfur - S 2p, for a sample of arsenopyrite prior to any treatments to remove surface oxidation. **(D)** Arsenic- As 3d, **(E)** iron- Fe 2p, and **(F)** sulfur- S 2p, for a sample of arsenopyrite treated with 12N HCl to remove surface oxides.

Representative curve-fitted XPS spectra for a sample prior to treatment are shown in Figure 2 (A-C). Excellent fits of the overall As 3d spectra (Figure 2A) could be obtained using a minimum of three peak doublets (3d_5/2_ and 3d_3/2_ separated by 0.64 eV) corresponding to two types of oxidized arsenic species along with unoxidized arsenic. The oxidized species with 3d_5/2_ binding energy 45.1 eV is most likely As^5+^, while the smaller doublet with a 3d_5/2_ binding energy of 43.9 eV is between the values typically reported for As^1+^ and As^3+^[1]. The Fe 2p_3/2_ spectra were also fitted with 2 peaks, corresponding to oxidized and unoxidized Fe (Figure 2B). No attempt was made to resolve the broad peak assigned to oxidized Fe into components that could be attributed to individual oxidation states since our only concern was the relative amounts of oxidized and unoxidized Fe. The S 2p spectra were resolved into three sets of doublets (Figure 2C) corresponding to the unoxidized sulfide (S 2p_3/2_ = 162.0 eV), a metal-deficient sulfide (S 2p_3/2_ = 163.2 - 163.7 eV), and oxidized sulfur in the form of sulfate (S 2p_3/2_ = 167.7 eV).

The reported percentages of oxidation were calculated based solely on the fraction of each element that is bound to oxygen. While the extents of oxidation measured from the Fe 2p_3/2_ and As 3d spectra for a given sample agreed within experimental error, the degree of sulfur oxidized to sulfate measured from the S 2p spectra for the same sample was much less. This has been commonly attributed to the formation of a metal-deficient sulfide species in other studies of the oxidation of pyrite and arsenopyrite surfaces [10],[13],[14]. Further, sulfur on the arsenopyrite surface, oxidizes at a rate much lower than iron and arsenic [11],[15], thereby resulting in a lower extent of sulfur oxidation on the surface.

The results in Table 1 clearly indicate that the drying procedure at room temperature is preferable to drying at 105°C as it limits the extent of surface oxidation. Thus, this procedure was implemented for drying the samples prior to applying various treatments to remove surface oxidation.

#### Removal of surface oxide species

**Figure 3:**
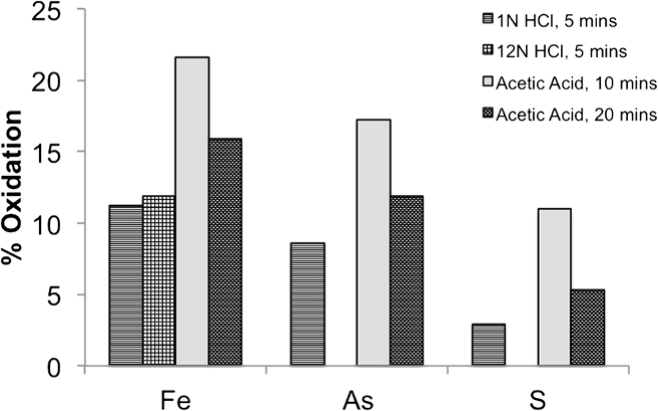
**Percentages of Fe, As, and S oxidation determined from XPS spectra of arsenopyrite after various cleaning procedures.**

The percentages of oxidation of each element determined from peak fitting the XPS spectra of the arsenopyrite samples after the application of various cleaning methods being tested to remove oxidized surface species are shown in Figure 3. The corresponding XPS spectra for the sample cleaned with HCl are shown in Figure 2D-2F.

The results indicate that treatment with the strong acid HCl was more effective in removing surface oxidation than treatment with the weak acid, acetic acid. A higher concentration of HCl was more effective in removing the last traces of surface oxidation over the given treatment time. Within the acetic acid treatments, increasing the treatment time resulted in increased removal of oxidized surface species, but even a treatment time of 20 minutes was insufficient for complete removal of oxidized species.

Only 12N HCl removed all oxidized species of sulfur and arsenic over the given treatment time. However, all four methods failed to remove oxidized iron completely, with 12N HCl being the most effective. For the case of 12N HCl, fitting of the Fe 2p_3/2_ spectra still indicated some residual surface oxidation. The presence of oxidized iron species has also been observed on vacuum fractured surfaces of arsenopyrite by Nesbitt et al. [11], who reported that ~17% of the Fe on the surface could be attributed to Fe (III)-(As-S) species. Moreover, the overall higher surface sensitivity of the XPS measurement to the Fe 2p level compared to As 3d and S 2p and/or some small amount of preferential reoxidation of Fe during the sample's exposure to air during its transfer to the XPS instrument could have also resulted in the detection of oxidized iron species, even after cleaning the surface. Comparing the S 2p spectra before and after the HCl treatment, one can see that while all the sulfur oxidized to sulfate has been completely removed by the treatment, the relative percent of metal-deficient polysulfide increased from 5% to 17% of the non oxygen-bound sulfur species. Although metal deficient polysulfide species have been reported in vacuum fractured arsenopyrite [1], oxidation of samples during procurement and grinding could have resulted in the formation of polysulfides on the surface [10],[13],[14].

While the 12N HCl cleaning method can be used for other sulfide minerals, using 12N HCl might not be appropriate for acid volatile sulfides (AVS) such as galena (PbS), which dissolve in concentrated HCl [16].

### SEM-EDS

**Figure 4:**
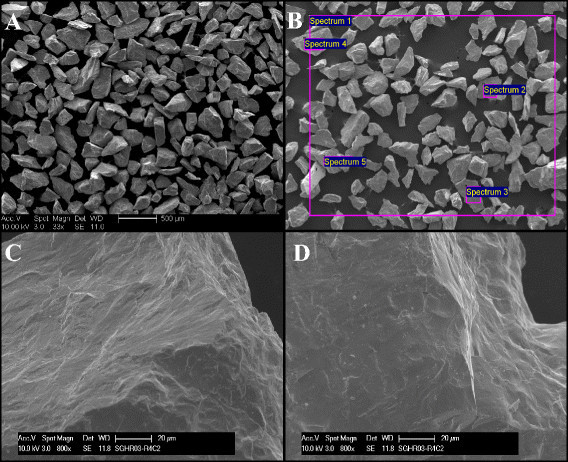
**SEM analysis of arsenopyrite particles. (A)** Secondary electron image of clean size segregated arsenopyrite particles **(B)** Sample surface composition measurements of arsenopyrite using SEM-EDS. Comparison of SEM images between **(C)** arsenopyrite surface prior to acid treatment and **(D)** arsenopyrite surface after cleaning with 12N HCl reveals no significant morphological changes to the surface of the mineral.

SEM images of freshly prepared arsenopyrite particles, cleaned with 12N HCl and 50% ethanol are shown in Figure 4. The ground mineral particles are uniformly sized and the surfaces do not show the presence of any fine particles (Figure 4A). The arsenopyrite particles were analyzed for iron, arsenic and sulfur using EDS (Figure 4B) and the measured weight percentages were converted to stoichiometric quantities. The prepared arsenopyrite sample was divided into ten batches and five measurements (Figure 4B) were made per batch. The average stoichiometry was found to be Fe_1.04±0.08_As_0.96±0.05_S_1.03±0.04_. Considering that sulfur on arsenopyrite oxidizes at a rate lower than Fe and As [11],[15], the surface is expected to be sulfur enriched after cleaning with HCl. While XPS measurements indicated some enrichment, the average mineral stoichiometry measured by SEM-EDS was close to the theoretical stoichiometry of FeAsS. Surface sulfur enhancement measured through XPS can be included in dissolution rate calculations while conducting dissolution experiments to ensure accurate measurement of rates of Fe, As, and S release.

Further, SEM images of arsenopyrite particles before (Figure 4C) and after treatment with 12N HCl (Figure 4D) did not reveal any significant morphological changes on the surface suggesting minimal change in the specific surface area of the mineral particles due to acid treatment. A comparison between the images depicts an absence of pitting or etching from acid treatment.

### Experimental

#### Mineral preparation and reagents

Arsenopyrite from Hunan province in China was obtained from Wards Science Inc. (Rochester, NY) in 10 g batches. Each batch was ground to powder using a porcelain mortar and pestle. The mortar and pestle were soaked with 10% HNO_3_ (w/v) overnight prior to being used for the first time. The powdered arsenopyrite was then dry sieved using 250 μm and 150 μm nylon sieves, which were also soaked in 10% HNO_3_ (w/v) overnight. The fraction of particles between 150-250 μm was collected and transferred into a plastic tube. From every 10 g batch of mineral, 5 g in the 150-250 μm fraction was obtained in this manner.

ACS reagent grade acids (HCl, acetic acid) were used in all cleaning experiments. All aqueous solutions were prepared using ultra-pure water (18.2 MOhm.cm, Barnstead Nanopure purifying system). Laboratory grade nitrogen (99.9% purity) was used in all experiments for mineral drying.

### Surface cleaning

Surface cleaning of arsenopyrite consisted of two steps:

1. Removal of fine particles adhered to the surface: particles significantly smaller than the sieved range can be electrostatically bound to the surface of larger particles. Such fines could result in exaggerated measurement of dissolution rates and hence need to be removed.
  To remove fines, the particles were sonicated with 50% (v/v) ethanol for 3 minutes using a Branson 5200 sonicator (Branson Inc., Connecticut, USA). Upon sonication, the ethanol phase turned black in color indicating suspended fine particles, and was subsequently decanted. This process was repeated three times and the particles were transferred to a petri dish and dried. Since drying the particles could affect the extent of surface oxidation, two different methods of drying were evaluated: a) drying the particles in air at 105°C for 15 minutes and b) drying the particles in air for 1 hour at room temperature (25°C). The dried arsenopyrite particles were then subjected to XPS analysis and a method for drying was chosen based on the extent of surface oxidation. Limiting the extent of initial surface oxidation enables easier removal of oxidized layers from the surface.
2. Removal of oxide layers - limited surface oxidation can occur during crushing, sieving, and drying of arsenopyrite. This can impact initial dissolution rate determination, as the rate of oxide phase dissolution can be significantly different from that of arsenopyrite. Further, arsenopyrite dissolution being oxidative in nature could be affected by dissolved oxidized species.
  Removal of oxides to the largest possible extent aids in accurate initial rate measurements. Oxides on the surface were removed by washing the arsenopyrite particles with acid.

Four methods for surface oxide removal were evaluated to identify an effective protocol for surface cleaning. These methods are shown in Table 2. As indicated there, the methods were similar except for the acid employed in the first rinse step, which involved either HCl (1N or 12N) or 50% v/v acetic acid.

**Table 2:** **Methods for cleaning surface oxides on arsenopyrite particles**

Method	Particle size	1^st^Rinse	2^nd^Rinse	3^rd^Rinse	Drying environment	Drying duration	Temp.
1	150-250 μm	1N HCl (5 mins)	DI^a^ water (3 mins)	50% (v/v) ethanol (1 min)	N_2_	1 h	25°C
2	150-250 μm	12N HCl (5 mins)	DI water (3 mins)	50% (v/v) ethanol (1 min)	N_2_	1 h	25°C
3	150-250 μm	50% (v/v) acetic acid (10 mins)	DI water (3 mins)	50% (v/v) ethanol (1 min)	N_2_	1 h	25°C
4	150-250 μm	50% (v/v) acetic acid (20 mins)	DI water (3 mins)	50% (v/v) ethanol (1 min)	N_2_	1 h	25°C

^a^DI: Deionized.

After cleaning the particles, samples intended for XPS analysis were placed in glass vials, flushed with N_2_, crimped, and transported in vacuum containers capable of maintaining vacuum of 14.7" Hg for over 24h, (Desi-Vac 700 mL containers, Cole palmer, USA). All samples were analyzed the same day that the cleaning method was applied.

## Conclusions

A method for preparing size-segregated arsenopyrite particles, free of surface oxides for dissolution experiments was developed. A summary of the developed method is shown in Table 3. Arsenopyrite particles in the size fraction of 150-250 μm was obtained by sonication of suspensions of crushed particles in 50% v/v ethanol and verified by particle size distribution and SEM analysis. Four methods of cleaning the surface were investigated and the method involving 12N HCl and 50% ethanol was found to be the most effective. XPS analysis revealed the method succeeded in removing all oxide species of S and As on the surface, while only 12% of surface Fe remained oxidized.

**Table 3:** **Summary of arsenopyrite preparation method**

1	Arsenopyrite was crushed in a mortar and pestle. Dry sieved to obtain 150-250 μm size fraction.
2	Sonicated in 50% ethanol and supernatant decanted (thrice).
3	Dried under N_2_ at room temperature for 1 hour.
4	Rinsed with 12N HCl for 5 minutes, followed by water (3 minutes) and 50% ethanol (1 minute)
5	Dried under N_2_ for 1 hour.

## Methods

### Particle size distribution

The effectiveness of sonication in removing fines was analyzed by measuring the average diameter of the particles by laser diffraction at Particle Tech Labs (Illinois, USA). Ten grams of sonicated and dried arsenopyrite were shipped in a 15 mL centrifuge tube (Corning USA) for analysis. The particle diameter distribution was calculated on a % volume basis.

### XPS

XPS measurements were carried out using a PHI 5600ci instrument. The XPS instrument employed monochromatic Al Kα X-rays and the pass energy of the analyzer was 23.5 eV. The arsenopyrite powders were attached to the sample holder using double-sided adhesive, electrically-conductive tape. Percentages of elemental oxidation were calculated from the relative areas of component peaks after the overall peak envelope for a given XPS peak was fitted with the component peaks due to oxidized and unoxidized forms of the element. Elemental concentrations were calculated using sensitivity factors provided by the instrument manufacturer. XPS peak fitting analyses were accomplished using CasaXPS data processing software. Binding energies were referenced to the C 1s peak for adventitious carbon at 284.6 eV.

### SEM- EDS

SEM was used for visual confirmation of arsenopyrite particle size and to evaluate surface composition of the particles. Once a suitable method for cleaning was chosen based on XPS data, arsenopyrite was prepared fresh, stored under vacuum, as previously described, and subjected to analysis within 2 hours of cleaning. The SEM was performed using a Philips XL30 FEG scanning microscope equipped with an SE Everhart Thornley detector and an Oxford INCA EDS with full quantitative composition analysis. The operating conditions were, accelerating voltage 10 kV, spot size 3, and a working distance of 10 mm. The SEM-EDS had a detection limit of 1% by weight.

## Authors’ original submitted files for images

Below are the links to the authors’ original submitted files for images. Authors’ original file for figure 1 Authors’ original file for figure 2 Authors’ original file for figure 3 Authors’ original file for figure 4
