# Supplementary figures and images for: A method for preparation and cleaning of uniformly sized arsenopyrite particles
Source: Geochem Trans. 2014 Oct 11;15:14. doi: 10.1186/s12932-014-0014-9 (PMC4212175; doi:10.1186/s12932-014-0014-9)

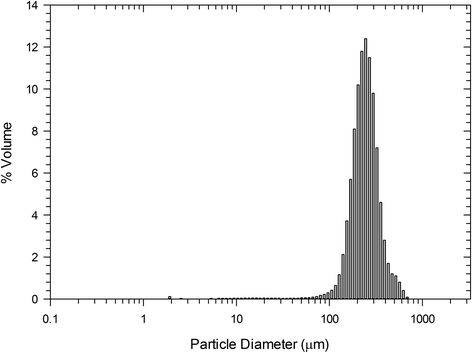

Supplement: Supplementary file 1 — Authors’ original file for figure 1 [file 12932_2014_14_MOESM1_ESM.gif]

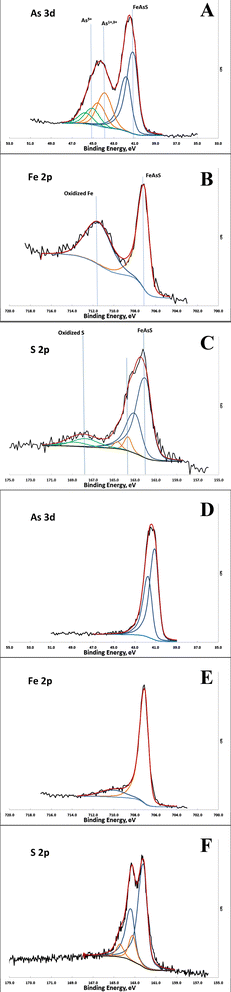

Supplement: Supplementary file 2 — Authors’ original file for figure 2 [file 12932_2014_14_MOESM2_ESM.gif]

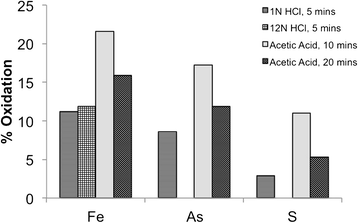

Supplement: Supplementary file 3 — Authors’ original file for figure 3 [file 12932_2014_14_MOESM3_ESM.gif]

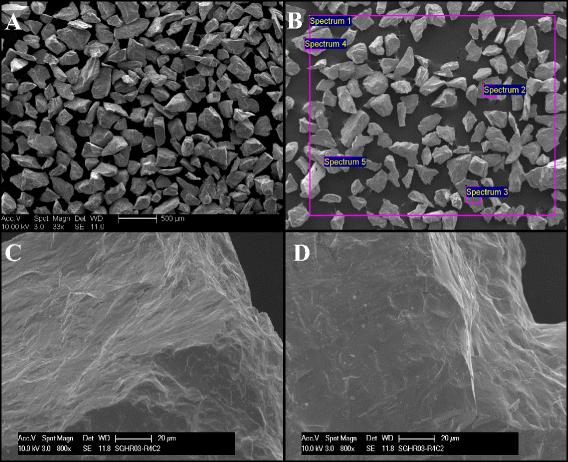

Supplement: Supplementary file 4 — Authors’ original file for figure 4 [file 12932_2014_14_MOESM4_ESM.gif]
